# The Association of Job Stress, Quality of Sleep, and the Experience of Near-Miss Errors among Nurses in General Hospitals

**DOI:** 10.3390/healthcare12060699

**Published:** 2024-03-21

**Authors:** Seong-Kyeong Kwak, Jin-Soo Ahn, Yeon-Ha Kim

**Affiliations:** 1 Graduation School of Nursing, Korea National University of Transportation, Jeungpyeong-gun 27909, Republic of Korea; kayinhau@naver.com; 2 Graduate School of Safety Engineering, Seoul National University of Science and Technology, Seoul 01811, Republic of Korea; 3 Department of Nursing, Korea National University of Transportation, Jeungpyeong-gun 27909, Republic of Korea

**Keywords:** near miss, healthcare, sleep quality, occupational stress, nurses

## Abstract

This study investigated the association of job stress, quality of sleep, and near-miss error experiences among nurses working in general hospitals. A convenience sample of 195 nurses with over 3 months of work experience in a general hospital participated in the study. Statistical analyses were performed using SPSS 27.0. Factors influencing experiences of near-miss errors were examined through univariable and multivariate logistic regression. In total, 58.5% of nurses in a general hospital had experienced near-miss errors. Nurses in the upper job stress tertile (≥118) were 2.24 times more likely to encounter near-miss errors (95% CI: 1.07–4.69) and particularly 2.58 times (95% CI: 1.26–5.26) in falls and medical equipment-related near-miss errors. Nurses working more than 3 h of overtime per week had a 2.72 times higher (95% CI: 1.35–5.48) likelihood of experiencing near-miss errors related to falls and medical equipment and 4.03 times higher (95% CI: 1.92–8.45) likelihood of experiencing near-miss errors associated with examination procedures. To prevent near-miss errors among nurses in general hospitals, effective management of organizational job stress is crucial. Particularly for departments with a high level of overtime work (more than 3 h/week), it is essential to provide and manage educational programs on patient safety.

## 1. Introduction

The complexity and intensity of work in the nursing environment have increased due to advancements in healthcare and an increased demand for medical services. As a result, there has been a rise in the incidence of near-miss errors—situations in which potential harm is identified during the delivery of nursing services [1]. Nevertheless, near-miss errors, which are also referred to as close-call incidents, represent preventable medical errors [2].

According to patient safety statistics in South Korea, 14,820 incidents have been reported from 2022 [3]. Near-miss errors constituted the largest category at 35.6%, followed by minor incidents at 26.9%, no harm events at 10.8%, deaths at 1.0%, and severe harm at 0.3% [3]. Specifically, in general hospitals, the number of near-miss errors rose from 2927 incidents in 2021 to 5283 in 2022, with a higher occurrence rate than in tertiary general hospitals [3]. Such near-miss errors can lead to negative outcomes, including increased healthcare costs, longer hospital stays, higher mortality rates, greater likelihood of readmission, and diminished trust in hospitals and healthcare professionals [4].

Identifying risk factors helps to understand near-miss errors, enabling the prevention of incidents and, more crucially, the avoidance of sentinel events that could endanger patient lives. Time pressure, concentration demands, and low job control increase stress [5] and cause adverse effects on an individual’s physical, social, and mental health. Higher stress levels increase the risk of human error, which highlights the importance of a risk management strategy. In nurses, job-related factors such as specific departmental experience [6], overtime hours [2], work schedules, and the frequency of night shifts [7] are known as risk working environment. Moreover, chronic and accumulated stress factors such as concentration demands and low job control have been linked to negative outcomes, including a reduction in patient safety activities [5]. Existing qualitative studies have indicated that stress from heavy workloads can lead to an uptick in medication near-miss errors [8,9]. A study stressed the importance of emotional and physical roles with a rise in medical errors in hospital nurses [10]. This underscores the critical need to further investigate the connection between nursing job stress and near-miss errors. Therefore, it is essential to understand the relationship between job stress and the occurrence of near-miss errors. However, despite its significance, to the best of our knowledge, no research to date has explored the relationship between nursing job stress and near-miss errors with a structured job stress tool designed for nurses.

Shift work in particular can disrupt circadian rhythms and lead to sleep disorders [7]. Nurses working on night shifts tend to suffer more frequent sleep problems, which has been identified as one cause of human error and can directly impact patient safety [11]. Accumulated poor sleep not only declines work efficiency but also increases the likelihood of making incorrect clinical judgments [11]; in turn, this heightens the frequency of errors and occupational incidents [10,12,13]. Therefore, it is necessary to examine nurses’ perceived quality of sleep associated with the near-miss errors as not all the near-miss errors result in accidents and are preventable [2]. However, the association between quality of sleep and near-miss errors has yet to be studied.

Previous studies have investigated the prevalence and influencing factors of near-miss errors in small-to-medium-sized hospitals [2,14] and in tertiary university hospitals [13]. Other studies conducted in small-to-medium-sized hospitals [8,15], as well as in tertiary university hospitals [13], have been confined to examining medication errors. However, research on near-miss errors among nurses in general hospitals remains insufficient. Therefore, it is necessary to explore not only medication errors but also a variety of near-miss incidents, such as pre- and post-examination procedures, falls, infection, verifying prescriptions, etc., related errors, and the factors that influence them. Furthermore, while there have been reports on the effects of sleep disorders on near-miss errors [13], the relationship between near-miss errors and patient safety culture [16], and the link between professional self-concept and near-miss errors [17], research is still lacking on the influence of job stress, sleep quality, and on the occurrence of near-miss errors.

Therefore, in this study, we aimed to assess the rate of near-miss error experiences and its related factors, as well as to examine the association of job stress, quality of sleep, and the experiences of near-miss errors among nurses in general hospitals. We hypothesized that general hospital nurses’ stressful work conditions/poor sleep condition would be related to near-miss error incidents.

Based on our findings, we hope to provide foundational information for the development of strategies to improve patient safety among nurses in general hospitals.

## 2. Materials and Methods

This descriptive survey-based study aimed to examine the association of job stress, quality of sleep, and the experience of near-miss errors among nurses in general hospitals.

### 2.1. Participants 

In total, 195 nurses working in a general hospital in Province C, South Korea, participated in this study. Based on previous literature and considering the adaptation period to the hospital [12], the inclusion criteria for the nurses were as follows: (1) at least 3 months’ work experience in a general hospital as a full-time job, (2) direct responsibility for the care of inpatients in wards and specialized departments, and (3) voluntary participation in the study. Nurses were excluded if they held roles focused on nursing administration and management. 

The sample size was calculated using the G*Power 3.1 software (Düsseldorf, Germany), based on a correlation analysis with an effect size of 0.2 (small), a power of 95%, a significance level of 0.05, and the inclusion of 14 predictor variables. This calculation also took into account an expected dropout rate of 20%. The minimum required sample size was determined to be 187 participants, which was considered sufficient for the study.

### 2.2. Measurements

A written self-report questionnaire was constructed to collect data on participants’ general characteristics, quality of sleep, job stress, and experience of near-miss errors. All tools were asked for permission to use or confirm verification from the author.

#### 2.2.1. Experience of Near-Miss Errors

The assessment of near-miss errors was conducted using an instrument originally developed by Park et al. [18] and later refined by Mun and Choi [13]. This instrument comprises 18 items divided into four categories: seven items for medication-related near-miss errors, six for protection-related, three for examination-related, and two for transfusion-related near-miss errors. Respondents indicate “yes” or “no” for each item based on their experiences over the previous four weeks. If they answer “yes,” they are further asked to quantify the frequency of these events, and the numbers were summed. As there was no validity information for the development and modification of this tool, this study evaluated the content validity, construct validity, and internal consistency of the instrument to assess nurses’ near-miss errors. Content validity was established by three nursing professors who evaluated the items and measurement methods, resulting in a content validity index of 0.96. Construct validity was determined through exploratory factor analysis. The Kaiser–Meyer–Olkin measure was 0.86, and Bartlett’s test of sphericity was statistically significant (chi-square = 1264.377, *p* < 0.001), indicating that the data were appropriate for factor analysis. Varimax rotation in the factor analysis identified four significant factors with loadings above 0.40, accounting for 60.00% of the variance. These factors were named according to the literature review: Factor 1 combined medication- and transfusion-related near-miss errors (7 items), Factor 2 included falls and medical equipment-related near-miss errors (2 items), Factor 3 comprised infection- and prescription-related near-miss errors (5 items), and Factor 4 consisted of examination-related near-miss errors (3 items). One item related to identifying and correcting an incorrect prescription was removed during the analysis, resulting in a revised tool with 17 items. Each item is scored on a binary scale (“yes” or “no”) based on the past four weeks’ experiences, with a “yes” response requiring further detail on event frequency. The numbers of frequency were summed, indicating that a higher reported frequency indicates a greater incidence of near-miss errors. The binary scale’s internal consistency was confirmed with a KR-20 of 0.83, and the frequency items showed a Cronbach’s α of 0.80.

#### 2.2.2. Quality of Sleep

The quality of sleep was measured using the Korean version of the Pittsburgh Sleep Quality Index (PSQI-K), which Cho [19] translated and validated based on the original instrument developed by Buysse et al. [20]. The PSQI-K assesses sleep quality over the preceding month and includes seven components. Each component is scored on a scale from 0 to 3, yielding a total score that ranges from 0 to 21. Higher scores signify worse sleep quality, and a score of 6 or above is indicative of poor sleep quality. The original instrument had a Cronbach’s α of 0.83, and upon translation by Cho [19], its reliability was reported to be 0.84. In the current study, the PSQI-K’s reliability was confirmed by a Cronbach’s α of 0.78, which is above the acceptable threshold of 0.60 as established by Nunnally [21].

#### 2.2.3. Job Stress

Job stress was measured using the Korean Nurses’ Occupational Stress Scale (K-NOSS), which was developed by Baek et al. [22]. This instrument includes various factors such as job demands—encompassing a hazardous working environment, physical, cognitive, and emotional job demands, roles and responsibilities, interpersonal conflict, shift work, work–life balance, and workplace violence—and job resources, which cover job autonomy, social support, organizational support, reward appropriateness, and organizational fairness. It consists of 45 items, each rated on a 4-point Likert scale, where 1 signifies “not at all”, 2 means “not really”, 3 corresponds to “yes”, and 4 indicates “absolutely yes”. The overall score can range from 45 to 180, with higher scores reflecting greater job stress. The original version of the tool showed a Cronbach’s α of 0.92, and in the current study, its reliability was confirmed by a Cronbach’s α of 0.89.

### 2.3. Data Analysis

All statistical analyses were conducted using SPSS version 23.0 (IBM Corp., Armonk, NY, USA). Descriptive data were analyzed using means ± SD and n (%). Sleep quality was classified as good or poor based on the cutoff point established by the measurement tool. Job stress factors were divided into upper, middle, and lower tertiles. To investigate differences in near-miss error experiences based on participants’ general characteristics, the *t*-test and one-way analysis of variance were utilized, with the Scheffé test conducted for post hoc analysis. The chi-square test was used to assess the association between participants’ general characteristics and the occurrence of near-miss error experiences. Both univariate and multivariate logistic regression analyses were carried out to identify factors that influence the incidence of near-miss errors.

### 2.4. Data Collection

Data collection took place from 11 September to 27 September 2023. The authors contacted the nursing department of the general hospital, informed them of the study’s purpose, and obtained approval from the nursing department. Before obtaining written consent from each participant, the authors informed the participants about the study’s purpose, methods, the assurance of anonymity and confidentiality throughout the research process, and their right to withdraw from the study at any time without any consequences. The Institutional Review Board (IRB) of the researchers’ affiliated university approved this study (IRB No. KNUT IRB 2023-15). The authors contacted each participant individually by phone to determine their eligibility based on the inclusion and exclusion criteria.

## 3. Results

### 3.1. Characteristics of the Participants and Differences in Near-Miss Error Experiences

The characteristics of the participants and the differences in near-miss error experiences based on these characteristics are presented in Table 1. 

The majority of the participants were women (174 individuals, 89.2%). The average age was 32.51 ± 7.67 years, with the highest number of participants (60 individuals, 30.8%) falling in the age range of 26 to 30 years. Participants’ average career experience was 6.81 ± 6.75 years, and 131 participants (67.2%) worked rotating shifts. The majority of participants held the position of acting staff nurse (157 individuals, 80.5%). The average number of night shifts was 5.03 ± 3.93 per month. The most common working department was the internal medicine ward, with 63 participants (32.3%). Furthermore, 21.5% of participants reported working more than 3 h of overtime per week. The majority of participants (46.2%) reported being responsible for 10 to 15 patients per day, and 93.8% of participants reported having received patient safety education.

Regarding near-miss error experiences based on general characteristics, participants who worked more than 3 h of overtime per week had a significantly higher average frequency of near-miss error experiences (5.90 ± 6.94) than those who worked less than 3 h (2.90 ± 5.81); this difference was statistically significant (t = −2.57, *p* = 0.013).

### 3.2. The Levels of Sleep Quality, Job Stress, Near-Miss Error Experiences, and the Rate of Near-Miss Error Experience

The levels of sleep quality, job stress, near-miss error experience, and the rate of near-miss error experiences among the participants are detailed in Table 2.

The total average score for quality of sleep was 9.51 ± 3.44. A poor quality of sleep stated by PSQI score ≥ 6 was present in 86.87% of subjects. 

Job stress had a total score of 110.44 ± 14.22, and by tertiles, the lower (≤104) was present in 34.9%, the middle (105–117) in 34.4%, and the upper (≥118) in 30.8% of subjects. Job stress had an average score of 2.45 ± 0.32; cognitive work demands scored the highest at 3.38 ± 0.65, followed by hazardous workplace environment at 2.87 ± 0.90, and emotional work demands at 2.81 ± 0.84. 

The mean number of near-miss error experiences of subjects was 3.55 ± 6.17. Among the sub-factors, experiences related to infection and prescription errors had the highest mean frequency (1.89 ± 4.43), while errors related to examinations had the lowest mean frequency (0.48 ± 0.13). The near-miss error experience rate was 58.5%. Examination-related errors had the highest occurrence rate, at 52.3%, followed by infection- and prescription-related errors (47.2%), falls and medical equipment-related errors (33.8%), and medication- and transfusion-related errors (20.5%).

### 3.3. Differences in Having Experienced a Near-Miss Error According to General Characteristics 

The results for differences in having experienced a near-miss error according to general characteristics among the participants are presented in Table 3. There was a statistically significant difference in job stress among the upper (≥118), middle (105–117), and lower (≤104) tertiles based on having experienced a near-miss error (χ^2^ = 6.23, *p* = 0.044).

### 3.4. The Association of Job Stress, Sleep Quality, and the Experience of Near-Miss Errors

The results of univariate (Step 1) and multivariate (Step 2) logistic regression analyses on the association of job stress, sleep quality, and the experience of near-miss errors are presented in Table 4.

Variables in general characteristics that showed significant differences in near-miss error experiences (overtime hours) and the main parameters (job stress and quality of sleep) were entered as independent variables. The presence of near-miss errors was set as the dependent variable in the logistic regression analysis.

In the univariate logistic regression analysis (Model 1), nurses with high job stress in the upper tertiles (≥118) were 2.24 times more likely to experience near-miss errors compared to those with lower levels of job stress (95% CI: 1.07–4.69). In the multivariate logistic regression analysis (Model 2), nurses with high job stress in the upper tertiles were 2.15 times more likely to experience near-miss errors than their counterparts without high job stress in the same tertiles (95% CI: 1.01–4.62). The results of the Hosmer–Lemeshow test showed no significant difference between observed and predicted values (χ² = 1.55, *p* = 0.818), confirming the model’s goodness of fit.

### 3.5. The Association of Job Stress, Sleep Quality, and Sub-Factors Related to Having Experienced a Near-Miss Error 

The results of univariate (Step 1) and multivariate (Step 2) logistic regression analyses assessing the association of job stress, sleep quality, and the sub-factors of near-miss error experiences are presented in Table 5.

No significant factors were identified that influenced near-miss errors related to medication and transfusion. For near-miss errors related to falls and medical equipment, the univariate logistic regression analysis (Model 1) showed that high levels of job stress (specifically, in the upper tertiles; ≥118) increased the likelihood by 2.11 times (95% CI: 1.01–4.39), and working overtime for more than 3 h per week increased the likelihood by 2.72 times (95% CI: 1.35–5.48). In the multivariate logistic regression analysis (Model 2), working overtime for more than 3 h per week increased the likelihood by 2.51 times (95% CI: 1.22–5.16).

For near-miss errors related to infection and prescription, the univariate logistic regression analysis (Model 1) showed that high levels of job stress (the upper tertiles; ≥118) increased the likelihood by 2.58 times (95% CI: 1.26–5.26). In the multivariate logistic regression analysis (Model 2), experiencing high levels of job stress (the upper tertiles; ≥118) increased the likelihood by 2.38 times (95% CI: 1.13–5.01).

For near-miss errors related to examinations, the univariate logistic regression analysis (Model 1) showed that working overtime for more than 3 h per week increased the likelihood by 4.03 times (95% CI: 1.92–8.45). In the multivariate logistic regression analysis (Model 2), working overtime for more than 3 h per week increased the likelihood by 3.44 times (95% CI: 1.60–7.36).

## 4. Discussion

This study aimed to assess the rate of near-miss error experiences among nurses in general hospitals and to identify related factors. Specifically, it examined the association of job stress, sleep quality, and the occurrence of near-miss errors, with the ultimate goal of providing foundational data to develop strategies that improve patient safety. 

The current research is consistent in terms of risk assessment. The findings of this study revealed that 58.5% of Korean nurses in general hospitals had experienced near-miss errors. This is less than the rates previously reported for Korean nurses in small-to-medium-sized hospitals, which stood at 63.8% [2]; however, this exceeds the rate in tertiary university hospitals, which was 49% [18]. This coincided with the statistics report that occurrence rates of near-miss errors in general hospitals are higher than in tertiary general hospitals [3]. 

Furthermore, among the sub-factors of near-miss error experiences, errors related to examination were the most common, accounting for 52.3% of incidents. This was followed by errors related to infection and prescription at 47.2%, falls and medical equipment at 33.8%, and medication and transfusion errors at 20.5%. These findings align with certain aspects of earlier research [18], that nurses who worked in wards at Korean tertiary university hospitals and experienced near-miss errors in the examination process were highest (71.4%), followed by patient protection in falls and ulcer infection (62.0%), finding an incorrect prescribed doctors order (59.2%), drug administering process (48.3%), and transfusion (3.5%). These findings contrast with certain aspects of previous study focusing on critical care nurses, which identified medication errors as the most frequent [13]. Nurses working in wards made errors more frequently than those who worked in another department [10]. Considering that the majority of participants in the current study were nurses from medical, surgical, and comprehensive nursing care service wards, this indicates a need for targeted education on preventing examination-related errors for all nurses who are directly responsible for inpatient care. 

However, most previous studies [8,9,15,18,23,24] with nurses focused more on medication errors. This indicated that the importance of near-miss errors related to pre- and post-examination procedures, falls and infection controls, and verifying prescriptions are underestimated and need to be emphasized. Nurses experienced near-miss errors that are preventable when having well-established reporting systems in hospitals [18]. Therefore, to reduce the rate of near-miss errors among general hospital nurses, it is necessary to develop a systematic reporting protocol for them to report errors. Moreover, supportive feedback is also needed, not to point out who was at fault but to provide a chance to share experiences to prevent errors from reoccurring. However, errors occur frequently during medication-related tasks [10].

The results of this study indicated that job stress was a significant factor influencing the occurrence of near-miss errors among nurses in a general hospital. Nurses who reported job stress at the upper tertiles level (≥118) were 2.24 times more likely to report near-miss errors (95% CI: 1.07–4.69). This association persisted even after adjusting for variables such as overtime hours and quality of sleep, with the likelihood of near-miss errors remaining 2.15 times higher for those experiencing job stress at the upper tertiles level (95% CI: 1.01–4.62). Within the sub-factors of job stress, factors in the job demand domain showed high scores; cognitive work demands were highest, followed by exposure to hazardous workplace environments and emotional work demands. Although direct comparisons are limited due to the scarcity of research specifically on job stress and near-miss errors, these findings are consistent with previous study that nurses with high emotional and bodily stress due to excessive workload cause near-miss errors to occur 4.65 times and 2.28 times more frequently than those with low emotional and bodily stress, respectively [10]. Previous studies stressed that job stressors and low job control are risk factors for patient safety [8], which is a concern with medical errors and mistakes [14]. As this study measured job stress with a structured tool tailored for nurses, the results are meaningful since no research to date has explored the relationship between nursing job stress and near-miss errors of general hospital nurses. Therefore, we conclude that nurses’ high-level job stress (≥118) affects the occurrence of near-miss errors and implies that the improvement of the working conditions of nurses is needed. Consequently, organizational management strategies, such as increasing staff numbers, regulating standard working hours, and improving working conditions, are necessary to mitigate excessive workload and enhance the work environment [6]. Previous study has suggested that for nurses who do become highly stressed, it should be considered to divide a hazardous work environment that leads to near-miss errors and consider temporarily transferring them to departments (e.g., outpatient) at lower risk of causing errors [10].

We found that overtime hours did not significantly affect near-miss error experiences. This finding is in contrast to a previous study that focused on nurses in small- and medium-sized hospitals [2], which found that nurses working more than 3 h of overtime per week had a 2.48 times greater likelihood of experiencing near-miss errors. However, our study results showed that nurses working in excess of three hours of overtime per week did have a significantly higher level of near-miss error experiences, aligning with certain aspects of earlier research [25] that indicated an increase in medication errors when overtime exceeded four hours. Previous study reported that nurses’ long work hours related with job stress [26]. Since nurses’ job stress was a significant factor influencing the occurrence of near-miss errors, long work hours might be a potential factor influencing the occurrence of near-miss errors. Consequently, additional research is warranted to resolve this inconsistency and to further examine the effects of extended work hours on near-miss errors. 

Upon examining the sub-factors of near-miss error experiences, it was found that nurses experiencing high job stress (a score of 118 or higher) had a 2.11-fold increased likelihood (95% CI: 1.01–4.39) of encountering near-miss errors related with falls and medical equipment. Additionally, they had a 2.58-fold increased likelihood (95% CI: 1.26–5.26) of encountering near-miss errors related to infections and prescriptions. Even after adjusting for overtime and sleep quality, nurses with high job stress (a score of 118 or higher) were still 2.38 times more likely (95% CI: 1.22–5.86) to experience near-miss errors pertaining to infections and prescriptions. These sub-factors include errors in nursing practices that are crucial for patient protection, such as preventing skin damage and infections and verifying prescriptions. Thus, managing job stress is essential for the prevention of near-miss errors in patient care. Previous studies on the quality of nursing care in long-term care hospitals have highlighted the importance of managing nursing staff burnout. This is considered an organizational indicator for the prevention of pneumonia and pressure ulcers in such units [27]. Consequently, to reduce the incidence of near-miss errors related to infections and prescriptions, it is necessary to develop indicators for job stress among nurses in general hospitals.

In this study, nurses who worked more than 3 h of overtime per week were found to be 2.72 times more likely to experience near-miss errors related to falls and medical equipment (95% CI: 1.35–5.48) and 4.03 times more likely to make errors related to examinations (95% CI: 1.92–8.45). These elevated risks persisted even after adjusting for job stress and quality of sleep, with the likelihood of experiencing these near-miss errors remaining at 2.51 times (95% CI: 1.22–5.16) and 3.44 times (95% CI: 1.60–7.36), respectively. Consequently, working in excess of 3 h of overtime per week may be a significant indicator of increased risk for near-miss errors concerning falls, medical equipment-, and examination-related errors among nurses in a general hospital setting. This underscores the necessity of implementing educational programs and management strategies for patient safety in departments where nurses frequently work overtime, with a focus on preventing patient falls, ensuring the safety of medical devices, and avoiding errors during examination procedures. Although direct comparisons are scarce due to the limited amount of prior research on near-miss errors specifically involving falls, medical equipment, and pre- and post-examination procedures, a study by Jin and Ha [28] indicated that the working hours of nurses affected fall prevention activities in university hospitals. The same study also noted that in general hospitals, extended working hours among nurses were associated with a decrease in fall prevention activities. Therefore, to reduce near-miss errors related to falls, medical equipment, and examinations among nurses in general hospitals, organizational measures such as ensuring adequate staffing and expanding flexible working hours are essential to improve working conditions [6]. Such measures can help to reduce the incidence of near-miss errors and enhance overall patient safety in general hospital environments. 

In this study, 86.7% of general hospital nurses showed poor sleep quality; however, this was not associated with near-miss errors or their sub-factors. This result is in contrast to earlier studies that have found poor sleep quality to be a contributing factor to medication-related near-miss errors and to an increased frequency of errors in transfusion and testing [13]. Although the same measurement tool was used, it is important to note that the previous study concentrated on specific departments, such as the emergency room and intensive care unit. In contrast, the majority of participants in the current study were nurses working in wards, and no significant differences in the occurrence of near-miss errors based on the work department was found. A study of Japan reported that nurses working in wards made errors more frequently than those who worked in another department [10]. Considering the substantial body of research suggesting a link between sleep quality and near-miss errors, further investigation is warranted to identify the reasons for this discrepancy and to clarify the relationship between sleep quality and near-miss errors in a broader context. 

The present study had several limitations. The first pertains to sample selection, as the survey was conducted among nurses in a specific general hospital. This may limit the generalizability of the research results to a broader population. Secondly, while we utilized a tool for near-miss errors, it is dependent on self-reports. It should be noted that the rate of near-miss errors reported by general hospital nurses may be affected by their subjective perceptions, which could lead to underreporting [13]. There is concern that recall bias among participants or potential biases in reporting errors could affect the objectivity of the data. This indicates a need for trained observers to rate with more objective and specific measurement tools to gather detailed information about near-miss errors. 

## 5. Conclusions

This study holds significant value, as it offers fundamental data that will inform efforts to promote patient safety in general hospitals. This study is meaningful since no research has explored the relationship between nursing job stress and near-miss errors with a structured job stress tool designed for nurses.

This study found that the rate of near-miss error experiences among Korean nurses in general hospitals was 58.5%. Although nurses previously focused more on medication errors, the examination-related near-miss errors were the most common among Korean nurses in general hospitals. Therefore, pre- and post-examination procedures need to be emphasized to prevent near-miss errors.

High levels of job stress increase the likelihood of encountering near-miss errors, particularly those related to infection and prescription. Therefore, special monitoring and organizational management are needed for nurses in general hospitals who experience high levels of job stress to prevent such near-miss errors.

Additionally, working 3 h of overtime per week increases the likelihood of experiencing near-miss errors related to falls, as well as medical equipment and examinations. This suggests that working over 3 h of overtime per week can be considered a risk indicator for nurses in general hospitals with respect to experiencing such near-miss errors.

To address these concerns, educational programs should be implemented that focus on patient infection prevention, fall prevention, medical equipment safety, and the prevention of prescription and examination errors. These programs are particularly crucial for departments where nurses work more than 3 h of overtime per week and experience high levels of job-related stress.

## Figures and Tables

**Table 1:** Characteristics of the participants and differences in near-miss error experiences (N = 195).

Variable	Category	n (%), Mean ± SD (Min–Max)	Near-Miss Error Experiences		*p* (Scheffé)
			Mean ± SD	t or F	
Gender	Male	21 (10.8)	3.19 ± 5.83	−0.30	0.770
	Female	174 (89.2)	3.60 ± 6.23		
Age (years)	<26	37 (19.0)	3.76 ± 8.39	0.32	0.813
	26–30	60 (30.8)	3.67 ± 5.92		
	31–35	42 (21.5)	2.74 ± 4.29		
	≥36	56 (28.7)	3.89 ± 6.02		
		32.51 ± 7.67 (22–56)			
Education	Diploma	32 (16.4)	4.16 ± 5.28	0.59	0.555
	Bachelor’s	148 (75.9)	3.29 ± 6.16		
	≥Master’s	15 (7.7)	4.80 ± 7.99		
Working career	<1	46 (23.6)	2.86 ± 4.37	0.48	0.700
(years)	1–4	58 (29.7)	3.34 ± 7.33		
	5–9	42 (21.5)	4.46 ± 6.95		
	≥10	49 (25.2)	3.75 ± 5.68		
		6.81 ± 6.75 (0.4–30.0)			
Working pattern	Day work	42 (21.5)	3.76 ± 5.95	0.57	0.568
	2 or 3 shifts	131 (67.2)	3.70 ± 6.60		
	Fixed (day or night)	22 (11.3)	2.23 ± 3.28		
Position	Staff nurse	157 (80.5)	3.47 ± 6.27	−0.40	0.689
	≥Charge nurse	38 (19.5)	3.89 ± 5.82		
Night shifts	<5	70 (35.9)	3.26 ± 5.24	0.13	0.874
(days/month)	5-7	78 (40.0)	3.64 ± 6.96		
	≥8	47 (24.1)	3.83 ± 6.18		
		5.03 ± 3.93 (0–16)			
Department	Intensive care	30 (15.4)	3.77 ± 4.99	0.76	0.520
	Internal medicine	63 (32.3)	3.57 ± 6.46		
	Surgical	52 (26.7)	2.58 ± 3.32		
	Comprehensive nursing care service	50 (25.6)	4.40 ± 8.36		
Overtime work hours	<3	153 (78.5)	2.90 ± 5.81	−2.57	0.013
(hours/week)	≥3	42 (21.5)	5.90 ± 6.94		
		1.51 ± 2.52			
Number of patients in	<10	53 (27.2)	3.69 ± 4.75	0.55	0.580
charge (per day)	10–15	90 (46.2)	3.09 ± 6.64		
	≥16	52 (26.7)	4.19 ± 6.65		
		12.31 ± 4.67 (7–17)			
Experience of patient	No	12 (6.2)	4.08 ± 5.05	0.37	0.716
safety training	Yes	183 (93.8)	3.51 ± 6.24		

**Table 2:** Levels of sleep quality, job stress, near-miss error experiences, and the rate of near-miss error experience (N = 195).

Variable	Sub-Domains and Sub-Factors	Mean ± SD (Min–Max)	Experience Rate n (%)
Quality of sleep		9.51 ± 3.44 (2–18)	
	Good (PSQI < 6)		26 (13.3)
	Poor (PSQI ≥ 6)		169 (86.7)
Job stress	Total (45–180)	110.44 ± 14.22 (68–145)	
Tertiles	Low(≤104)		68 (34.9)
	Medium (105–117)		67 (34.4)
	High (≥118)		60 (30.8)
Sub-domain	Average score (1–4)	2.45 ± 0.32 (1–4)	
Job demands	Hazardous workplace environment	2.87 ± 0.90	
	Physical work demands	2.56 ± 0.87	
	Cognitive work demands	3.38 ± 0.65	
	Emotional work demands	2.81 ± 0.84	
	Roles and responsibilities	2.47 ± 0.82	
	Relationship conflict	2.02 ± 0.71	
	Work schedule	2.37 ± 0.79	
	Work–life balance	2.36 ± 0.81	
	Workplace violence	2.01 ± 0.92	
Job resources	Job autonomy	2.44 ± 0.75	
	Social support	1.89 ± 0.56	
	Organizational support	2.39 ± 0.71	
	Adequacy of compensation	2.59 ± 0.74	
	Organizational fairness	2.33 ± 8.40	
Experience of near miss	Total	3.55 ± 6.17 (0–32)	114 (58.5)
	Medication and transfusion	0.53 ± 1.35 (0–7)	40 (20.5)
	Falls and medical equipment	0.65 ± 1.22 (0–9)	66 (33.8)
	Infection and prescription	1.89 ± 4.43 (0–24)	92 (47.2)
	Examination	0.48 ± 1.13 (0–7)	102 (52.3)

**Table 3:** Differences in having experienced a near-miss error according to general characteristics (N = 195).

Variable	Category	Near-Miss Experience n (%)		χ^2^	*p*
		No	Yes		
Gender	Male	10 (12.3)	11 (9.6)	0.36	0.549
	Female	71 (87.7)	103 (90.4)		
Age (years)	<26	13 (16.0)	24 (21.1)	0.96	0.812
	26–30	27 (33.3)	33 (28.9)		
	31–35	18 (22.2)	24 (21.1)		
	≥36	23 (28.5)	33 (28.9)		
Education	Diploma	11 (13.6)	21 (18.4)	0.90	0.637
	Bachelor’s	63 (77.8)	85 (74.6)		
	≥Master’s	7 (8.6)	8 (7.0)		
Working career	<1	19 (23.5)	27 (23.7)	0.03	0.999
(years)	1–4	24 (29.6)	34 (29.9)		
	5–9	15 (18.5)	20 (17.5)		
	≥10	23 (28.4)	33 (28.9)		
Working pattern	Day work (9 a.m.–6 p.m.)	18 (22.2)	24 (21.1)	0.04	0.981
	2 or 3 shifts	54 (66.7)	77 (67.5)		
	Fixed (day or night)	9 (11.1)	13 (11.4)		
Position	Staff nurse	68 (84.0)	89 (78.1)	1.04	0.307
	≥Charge nurse	13 (16.0)	25 (21.9)		
Night shifts	<5	32 (39.5)	38 (33.3)	1.61	0.447
(days/month)	5–7	33 (40.7)	45 (39.5)		
	≥8	16 (19.8)	31 (27.2)		
Department	Intensive care	11 (13.6)	19 (16.7)	1.73	0.630
	Internal medicine	27 (33.3)	36 (31.6)		
	Surgical	19 (23.5)	33 (28.9)		
	Comprehensive nursing care service	24 (29.6)	26 (22.8)		
Overtime work hours	<3	68 (84.0)	85 (74.6)	2.47	0.116
(hours/week)	≥3	13 (16.0)	29 (25.4)		
Number of	<10	21 (25.9)	32 (28.1)	0.60	0.742
patients in charge	10–15	40 (49.4)	50 (43.8)		
(per day)	≥16	20 (24.7)	32 (28.1)		
Experience of	No	4 (4.9)	8 (7.0)	0.35	0.764 *
patient safety training	Yes	77 (95.1)	106 (93.0)		
Quality of sleep	Good (PSQI < 6)	11 (13.6)	15 (15.2)	0.01	0.932
	Poor (PSQI ≥ 6)	70 (86.4)	99 (86.8)		
Job stress	Low (≤104)	32 (39.5)	36 (31.6)	6.23	0.044
	Medium (105–117)	32 (39.5)	35 (30.7)		
	High (≥118)	17 (21.0)	43 (37.7)		

* Fisher’s exact test; PSQI, Pittsburgh Sleep Quality Index.

**Table 4:** The association of job stress, sleep quality, and the experience of near-miss errors (N = 195).

Variable	Model 1						Model 2					
	B	SE	Wald	OR	95% CI	*p*	B	SE	Wald	OR	95% CI	*p*
Overtime work hours (≥3 h/wk) *	0.57	0.37	2.43	1.78	[0.86–3.69]	0.119	0.42	0.38	1.24	1.53	[0.72–3.24]	0.265
Quality of sleep (poor) *	0.36	0.42	0.01	1.03	[0.45–2.39]	0.932	−0.09	0.44	0.04	0.91	[0.38–2.17]	0.834
Job stress (medium: 105–117) *	−0.02	0.34	0.01	0.97	[0.49–1.91]	0.935	−0.01	0.35	0.01	0.99	[0.51–1.97]	0.989
Job stress (high: ≥118) *	0.81	0.37	4.65	2.24	[1.07–4.69]	0.031	0.76	0.38	3.88	2.15	[1.01–4.62]	0.049

* reference: Overtime work hours (<3 h/wk), quality of sleep (good), job stress (low: ≤104). Model 1 = Univariable logistic regression; Model 2 = Multivariable logistic regression.

**Table 5:** The associated of job stress, sleep quality, and the sub-factors related to having experienced a near-miss error (N = 195).

Sub-Factors	Variable	Model 1						Model 2					
		B	SE	Wald	OR	95% CI	*p*	B	SE	Wald	OR	95% CI	*p*
Falls and medical equipment	Overtime work hours (≥3 h/wk) *	1.01	0.35	7.89	2.72	[1.35–5.48]	0.005	0.92	0.36	6.29	2.51	[1.22–5.16]	0.012
	Job stress (high: ≥118) *	0.74	0.37	3.97	2.11	[1.01–4.39]	0.046						
Infection and prescription	Job stress (high: ≥118) *	0.94	0.36	6.76	2.58	[1.26–5.26]	0.009	0.87	0.37	5.27	2.38	[1.13–5.01]	0.022
Examination	Overtime work hours (≥3 h/wk) *	1.39	0.37	13.66	4.03	[1.92–8.45]	<0.001	1.23	0.38	10.13	3.44	[1.60–7.36]	0.001

* reference: Overtime work hours (<3 h/wk), job stress (low: ≤104). Model 1 = Univariable logistic regression; Model 2 = Multivariable logistic regression.

## Data Availability

Data are contained within the article.
